# Impact of urbanization on gut microbiome mosaics across geographic and dietary contexts

**DOI:** 10.1128/msystems.00585-24

**Published:** 2024-09-17

**Authors:** Elizaveta Vinogradova, Nurislam Mukhanbetzhanov, Madiyar Nurgaziyev, Zharkyn Jarmukhanov, Rakhilya Aipova, Aliya Sailybayeva, Makhabbat Bekbossynova, Samat Kozhakhmetov, Almagul Kushugulova

**Affiliations:** 1 Center for Life Sciences, National Laboratory Astana, Nazarbayev University, Astana, Kazakhstan; 2 Kazakh Research Institute of Soil Science and Agricultural Chemistry named after U.Uspanov, Almaty, Kazakhstan; 3 JSC “National Research Cardiac Surgery Center”, Astana, Kazakhstan; University of Massachusetts Medical School, Brookline, Massachusetts, USA

**Keywords:** urbanization, gut microbiome, microbial diversity, dietary patterns, geographic patterns, antibiotic resistance, virulence factors

## Abstract

This study provides a comprehensive assessment of how urban-rural divides influence gut microbial diversity and composition across the distinct geographical landscapes of Kazakhstan, elucidating the intricate interplay between lifestyle, environment, and gut microbiome. In this prospective cohort study, we enrolled 651 participants from urban centers and rural settlements across Kazakhstan, following ethical approval and informed consent. Comprehensive demographic, dietary, and stool sample data were collected. 16S rRNA gene sequencing and shotgun metagenomics techniques were employed to delineate the intricate patterns of the gut microbiome. A rigorous statistical framework dissected the interplay between urbanization gradients, geography, dietary lifestyles, and microbial dynamics. Our findings demonstrate a stark microbial divide between urban and rural gut ecosystems. The study found significant differences in gut microbiome diversity and composition between urban and rural populations in Kazakhstan. Urban microbiomes exhibited reduced diversity, higher Firmicutes/Bacteroidetes ratios, and increased prevalence of genera *Coprococcus* and *Parasutterella*. In contrast, rural populations had greater microbial diversity and abundance of *Ligilactobacillus*, *Sutterella*, and *Paraprevotella*. Urbanization also influenced dietary patterns, with urban areas consuming more salt, cholesterol, and protein, while rural areas had diets richer in carbohydrates and fiber. The study also identified distinct patterns in the prevalence of antibiotic resistance genes and virulence factors between urban and rural gut microbiomes. This study sheds light on how urbanization may be deeply involved in shaping the intricate mosaic of the gut microbiome across Kazakhstan’s diverse geographical and dietary landscapes, underscoring the complex interplay between environmental exposures, dietary lifestyles, and the microbial residents inhabiting our intestines.

**IMPORTANCE:**

The study examined gut microbiome composition across diverse geographical locations in Kazakhstan, spanning urban centers and rural settlements. This allows for thoroughly investigating how urbanization gradients and geographic factors shape the gut microbiome. The study's examination of the gut resistome and prevalence of virulence-associated genes provide essential insights into the public health implications of urbanization-driven microbiome alterations. Collecting comprehensive demographic, dietary, and stool sample data enables the researchers to better understand the relationships between urbanization, nutritional patterns, and gut microbiome composition. The findings have important implications for understanding how urbanization-driven microbiome changes may impact human health and well-being, paving the way for tailored interventions to restore a balanced gut microbial ecology.

## INTRODUCTION

Urbanization, characterized by rapid population growth, increased industrialization, and lifestyle changes, significantly impacts the composition and diversity of the gut microbiome (1–4). Studies from different geographical regions have provided compelling evidence of how urbanization can influence the gut microbiota. For example, research in China suggests that urban populations have a microbiome, marked by reduced microbial diversity and an increased prevalence of virulence and antibiotic-resistant genes (5).

Interestingly, rural populations, particularly those adhering to traditional dietary patterns, have consistently shown greater diversity and stability in their gut microbiota compared to urban populations (6, 7). The shift toward a more Westernized diet has been identified as a significant contributor to the observed alterations in the gut microbiome (8, 9).

These patterns have been observed worldwide, including in China, the United States, Africa, South America, India, Malaysia, and Thailand (6, 10–18). Research has consistently highlighted the impact of urbanization on microbial diversity and composition. For example, research comparing rural Mongolians and urban Chinese revealed stark differences in gut microbial profiles, with urban populations demonstrating lower diversity and higher levels of pro-inflammatory bacteria (5). Rural populations demonstrated higher microbial diversity and an abundance of beneficial bacteria associated with fiber-rich diets (3, 11).

Kazakhstan, a country undergoing rapid urbanization, presents a unique opportunity to explore this relationship across diverse geographical locations. Therefore, we aimed to assess the impact of urbanization on gut microbial diversity and composition in different geographical locations of Kazakhstan and to understand the relationship between urban/rural living, geography, and the gut microbiome.

## RESULTS

### Core microbiome

**Fig 1:**
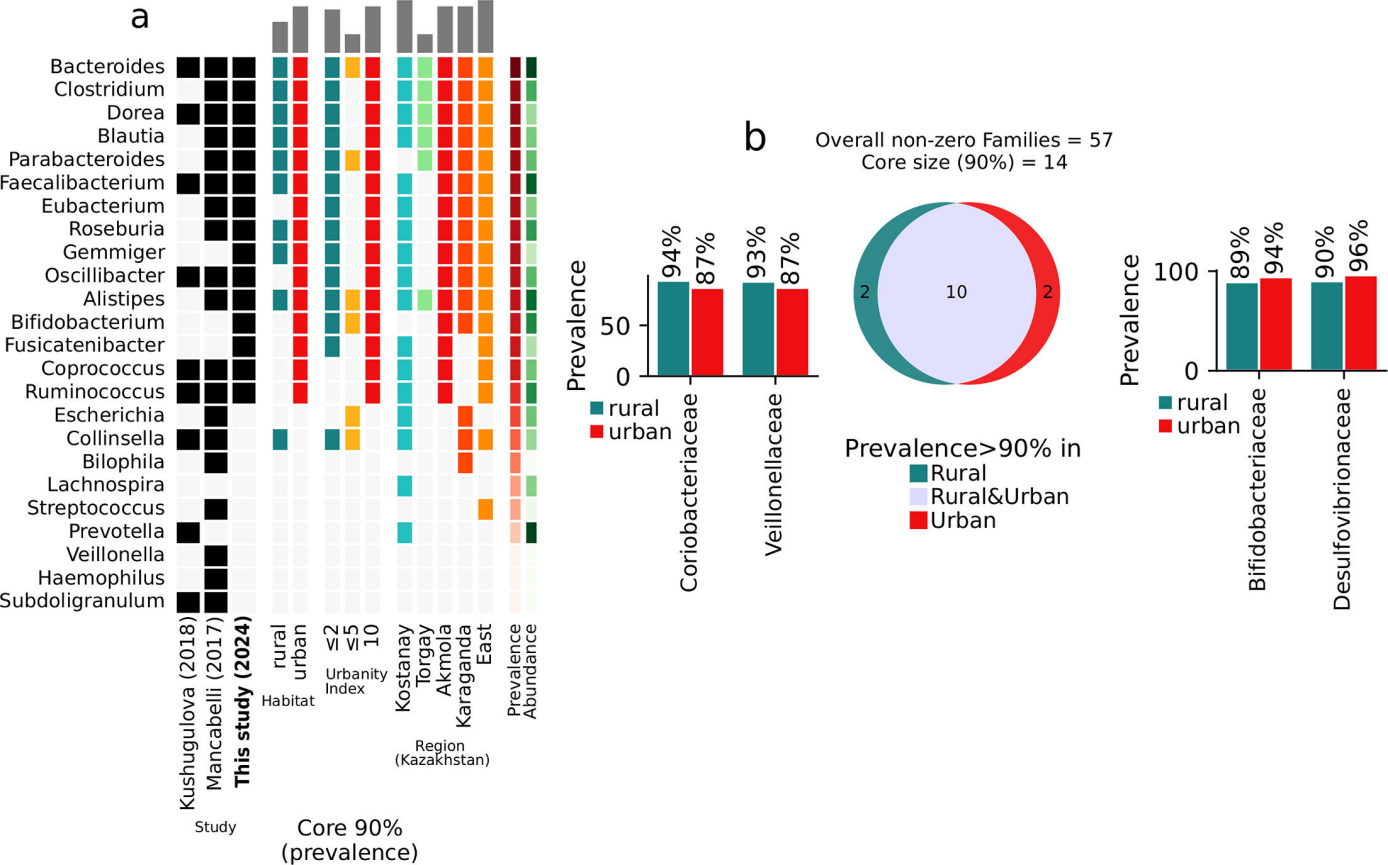
Core microbiome of the human gut. (a) Identified in meta-analysis conducted by Mancabelli et al. (20). Multicountry cohort, including agricultural and industrialized countries; in study by Kushugulova et al. (19). Kazakh population; in the present study, overall and in all subgroups, Kazakh population. Only genera present in at least 90% of all samples in the group are colored. (b) Core taxa overlap at the family level between rural and urban habitat samples and bar chart depicting prevalence of non-overlapping families.

By assessing the overall gut microbiome of Kazakhstanis and comparing it with our earlier study, the data reveal a high prevalence of several genera, including *Alistipes, Bacteroides, Bifidobacterium, Blautia, Clostridium, Eubacterium, Faecalibacterium, Gemmiger,* and *Prevotella*, with a prevalence of over 90%. Figure 1 demonstrated that most genera are shared with our previous data (19). Genera such as *Bacteroides, Dorea, Oscillibacter, Coprococcus, Ruminococcus,* and *Faecalibacterium* appear to be prevalent in both data sets. In contrast, others such as *Alistipes, Gemmiger, Clostridium, Parabacteroides, Eubacterium, Roseburia, Bifidobacterium,* and *Fusicatenibacter* show a higher prevalence in the current data. This is most likely due to the limitations of the previous study, which focused on the population of a large city; in this research, we analyzed the populations of several towns and small villages. There are also some differences when comparing the current data with the multipopulation data (second column) (20). Genera, such as *Gemmiger, Bifidobacterium,* and *Fusicatenibacter,* show a higher prevalence in the current data, while others like *Collinsella* appear more prevalent in the multipopulation.

The known characteristics of the microbiome representatives and their functional roles suggest a predominance of bacteria involved in the fermentation of dietary fibers and producing short-chain fatty acids (SCFAs). This is further supported by the predominant classification of Kazakhstanis into the Bacteroides enterotype (65% or 421 out of 651 samples were assigned to the Bacteroides cluster), which is characterized by bacteria proficient in breaking down complex polysaccharides from dietary fiber, leading to the production of SCFAs as metabolic by-products.

### Characteristics of the gut microbiome depending on the urbanity index

**Fig 2:**
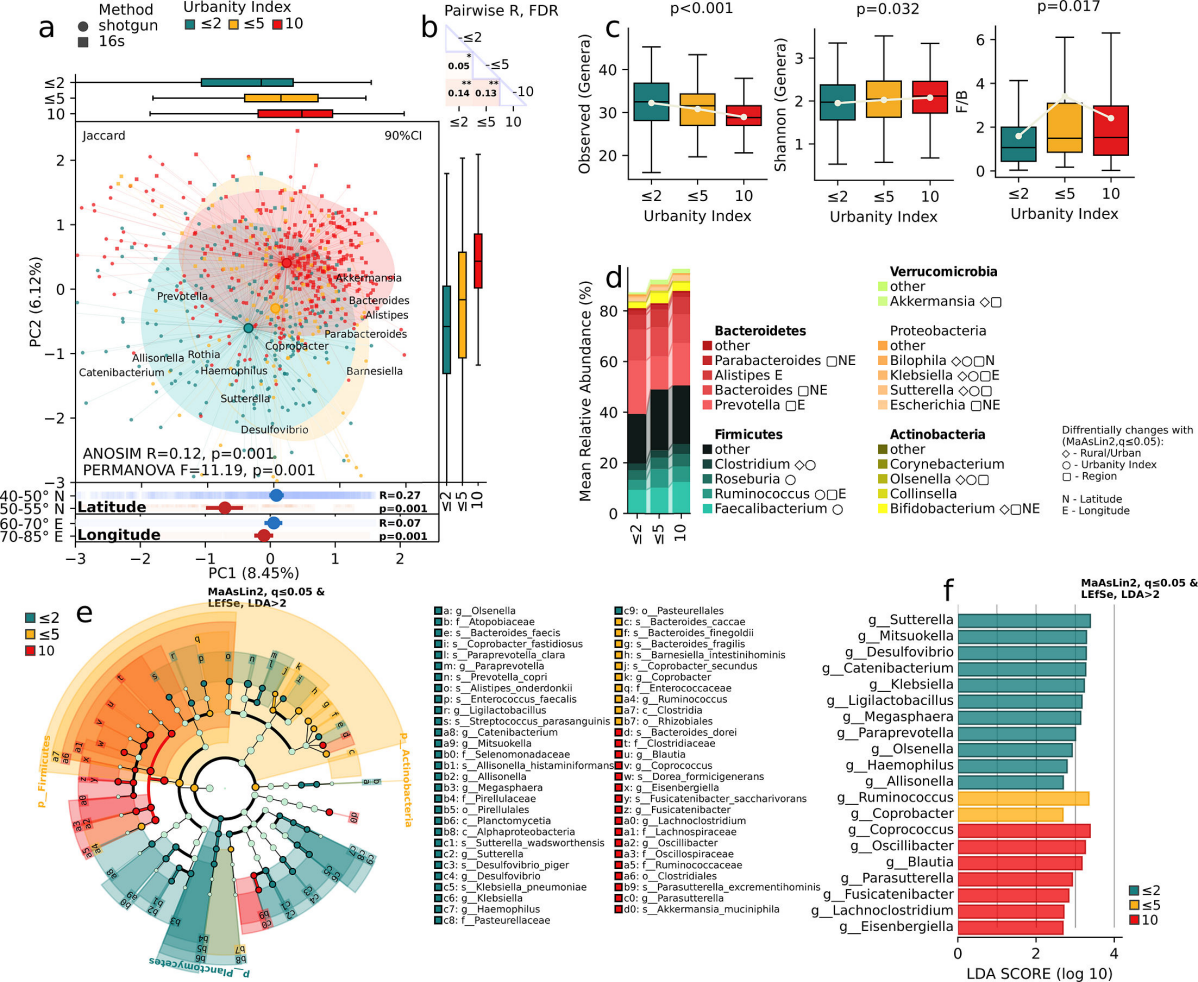
Biodiversity and taxonomic composition in relation to the urbanity index. (a) Principal coordinates ordination of the samples’ composition at the species level. Jaccard distance. (b) Groupwise test for difference in metagenomic composition. ANOSIM r, FDR. (c) Within-sample number of observed genera, Shannon index, and Firmicutes/Bacteroidetes ratio. Regression analysis, adjusting for demographic parameters, MaAsLin2, *P* ≤ 0.05. (d) Stacked bar chart displaying the relative abundance of the most abundant genera in the major bacterial phyla across urbanity index categories; MaAsLin2, *q* ≤0.05, FDR. (e) A cladogram depicting the differentially abundant bacterial taxa. (f) Bar chart depicting all identified differentially abundant genera. MaAsLin2, *q* ≤0.05, FDR, followed by LEfSe effect size analysis, LDA >2.

The findings of a study have revealed that urbanization exerts a discernible impact on the composition and diversity of the gut microbiota within human populations (Fig. 2c). We found significant differences in beta diversity indices, such as Bray-Curtis (*R* = 0.1, *P* = 0.001, *F* = 8.27, *P* = 0.001), Jaccard (*R* = 0.12, *P* = 0.001, *F* = 11.19, *P* = 0.001), W-Unifrac (*R* = 0.03, *P* = 0.003, *F* = 4.22, *P* = 0.002), and U-Unifrac (*R* = 0.1, *P* = 0.001, *F* = 11.11, *P* = 0.001), between gut microbiomes depending on the level of urbanization (Fig. 2a and b). The analysis of relative abundance, which quantifies the proportion of various taxa present, has unveiled noteworthy disparities across all taxonomic levels (Fig. 2d). This suggests that the differences observed in the gut microbiome composition among settlements of varying urbanization levels are not confined to specific taxa but manifest across the entire taxonomic hierarchy. This is reflected in the Firmicutes/Bacteroidetes (F/B) ratio, which increases with urbanization (*P* = 0.017).

The regression analysis, adjusting for demographic parameters, MaAsLin2 followed by LEfSe analysis, uncovered distinct gut microbiome composition features across varying urbanization levels (Fig. 2e and f). The gut microbiomes of individuals living in urban areas with an urbanization index of 10 (Astana city, the capital of Kazakhstan) were characterized by the family Lachnospiraceae, genera *Coprococcus* and *Parasutterella*, and species *Dorea formicigenerans*. Areas corresponding to an urbanization index of 5 exhibited a predominance of the *Coprobacter, Ruminococcus*, and species *Bacteroides coccae, Bacteroides finegoldii*, and *Bacteroides faecis* (Fig. 2e). Meanwhile, individuals residing in regions with an urbanization index below 3 displayed an intestinal microbiome profile characterized by representatives such as the order Pirellulales, families Pirellulaceae and Selenomonadaceae, and genera *Ligilactobacillus, Sutterella, Klebsiella, Catenibacterium, Desulfovibrio, Olsenella,* and *Anaerotruncus*, each exhibiting varying levels of dominance (Fig. 2f).

**Fig 3:**
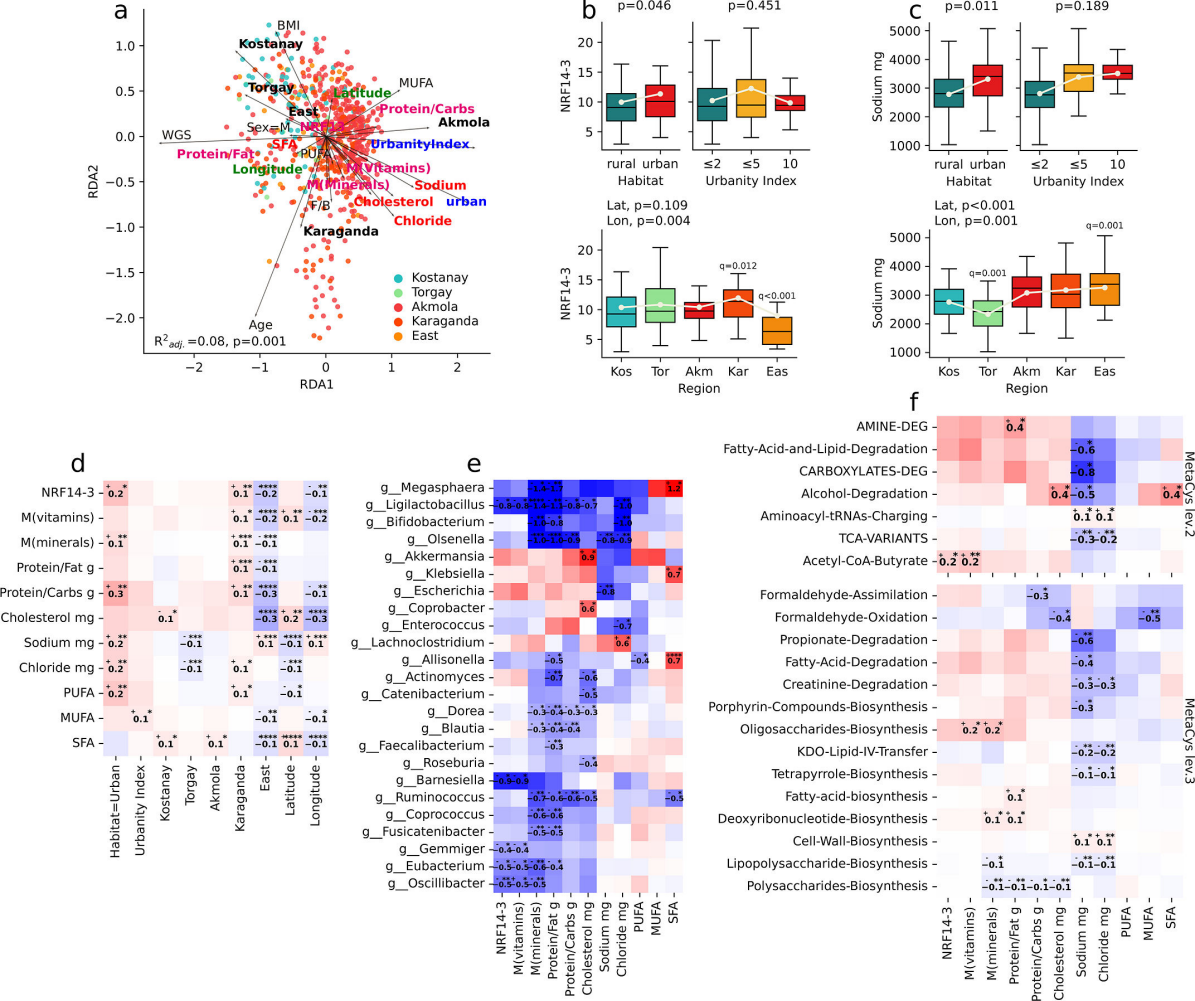
Association of bacterial taxa and metabolic pathways with diet and urbanization. (a)Redundancy analysis ordination with biplot visualizes the proportion of variation in taxonomic composition explained by the study variables. (b and c)Boxplots of the distribution of a dietary score (NRF14-3) and various nutrients/dietary components across rurality, urbanity index levels, and regions. (d)Associations between NRF14-3, nutrient intakes, and location (e)and differentially abundant genera (any subgroup); (f)metabolic pathways at levels 2 and 3 of the MetaCys hierarchy; regression analysis, adjusting for demographic parameters, MaAsLin2, *P* ≤ 0.05. Heatmaps display a regression coefficient that indicates the relative strength and direction of the association between variables.

Notably, these dominant genera (Fig. 2f) were found to be significantly correlated with the urbanization index in the MaAsLin2 analysis (FDR, *q* ≤0.05), in most cases exhibiting a high linear correlation. Nutrition data analysis revealed that urbanization impacts dietary patterns (Fig. 3a through d).

### Geographic influences on gut microbiome composition and dietary patterns

**Fig 4:**
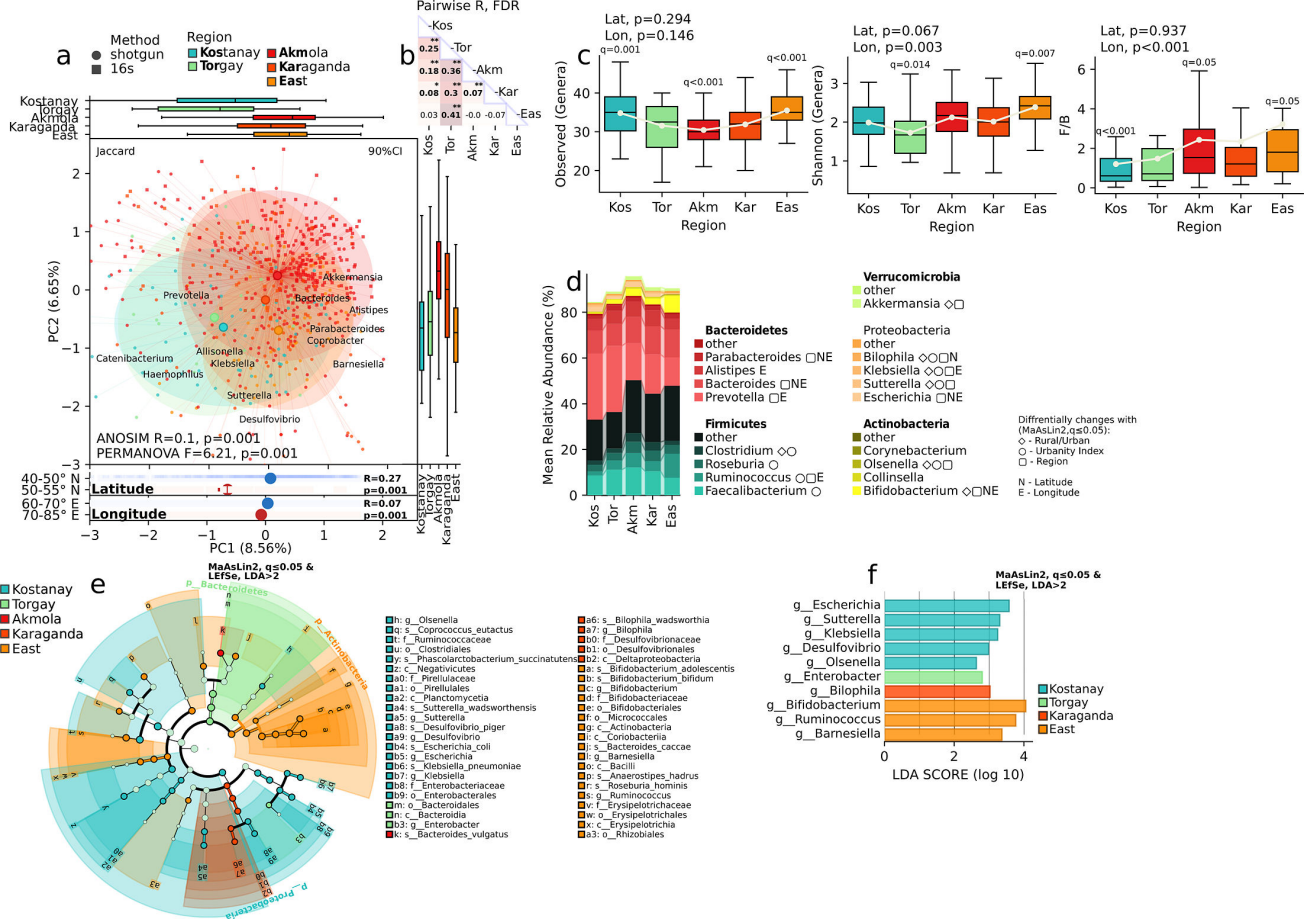
A comprehensive analysis of microbiome compositions across different geographic regions. (a) A principal coordinate analysis plot of the clustering patterns of samples by different geographic regions (Kostanay, Torgay, Akmola, Karagandy, and East Kazakhstan); (b) groupwise test for difference in metagenomic composition. ANOSIM r, FDR. (c) Boxplots of the observed genera richness among the regions. Observed genera, Shannon index, and Firmicutes/Bacteroidetes ratio. Regression analysis, adjusting for demographic parameters, MaAsLin2, *P* ≤ 0.05; (d) a stacked bar plot of the mean relative abundances of major bacterial phyla/taxa in the different regions, with annotations indicating differential abundances associated with rural/urban settings, urbanity index, and geographic location. MaAsLin2, *q* ≤0.05, FDR. (e) A cladogram of the taxonomic relationships; (f) a bar chart of the linear discriminant analysis (LDA) scores, which quantify the differential abundances of bacterial taxa associated with each geographic region. MaAsLin2, *q* ≤0.05, FDR, followed by LEfSe effect size analysis, LDA >2.

In addition to the urbanization gradient, we conducted analyses by latitude and longitude as well as within individual regions and identified significant differences that are reflected in alpha diversity (Fig. 4c), beta diversity (Fig. 4a and b), the relative abundance of bacteria at different taxonomic levels (Fig. 3d), and LEfSe analysis (Fig. 4e and f).

Latitude-related distinctions align closely with regional characteristics. LEfSe effect size analysis conducted after MaAsLin2 multivariable association analysis, has revealed unique taxonomic characteristics that define the gut microbiome of different populations in distinct regions while accounting for the possible influence of age, sex, and body mass index (BMI) variation on taxonomic composition. In Astana, an urbanized city, a distinct gut microbial composition is observed, characterized by a prevalence of the Clostridia family and the *Bacteroides vulgatus* species. In contrast, the major city of Karagandy exhibits dominance represented by the *Bilophila wadsworthia* species.

Meanwhile, the regional hub of Kostanay shows predominance at various taxonomic levels, including families such as Ruminococcaceae*,* Pirellulaceae, and Enterobacteriaceae*,* alongside genera *Suterella, Desulfovibrio, Klebsiella,* and *Escherichia*, as well as species *Phascolarctobacterium succinatutens* and *Coprococcus eutactus*. Similarly, the Torgai region is distinguished by an abundance of the Enterobacter genus. In contrast, the gut microbiome of the Eastern Kazakhstan populace is marked by a notable predominance at the family level of Erysipelotrichaceae and Bifidobacteriaceae*,* with dominance at the genus level observed in *Bifidobacterium* and *Ruminococcus*.

Regarding longitude, noteworthy differences emerged between Eastern and Central Kazakhstan: Eastern Kazakhstan is characterized by a prevalence of genera, such as *Bifidobacterium, Bacteroides, Ligilactobacillus,* and *Parabacteroides*, while genera *Prevotella, Faecalibacterium, Escherichia, Klebsiella,* and *Clostridium* predominate in Central Kazakhstan.

Our findings underscored the pronounced impact of urbanization on dietary constituents, notably revealing heightened consumption of salt (chloride and sodium) and cholesterol in urban settings (*P* = 0.0118 and *P* = 0.0138, and *P* = 0.2811). Conversely, sugar intake exhibited an inverse relationship with urbanization (*P* = 0.0112), with Astana demonstrating the lowest rates (*P* = 0.02). So, in our study, the NRF indicator, based on 14 beneficial nutrients and on 3 nutrients to limit, also increases with increasing urbanization (*P* = 0.046).

The Nutrient-Rich Foods (NRF) index is positively correlated with the acetyl-CoA-butyrate metabolic pathway (*P* = 0.033) (Fig. 3f), a short-chain fatty acid produced by gut microbiota from dietary fiber, which plays a key role in liver mitochondrial function and efficiency (21, 22).

Urban environments typically provide increased access to a wider range of fortified and diverse food options, potentially leading to higher M(mineral) (M = average of consumed/recommended ratios) among urban residents (*P* = 0.005). Urban populations exhibit a higher protein/fat ratio than their rural counterparts (*P* = 0.182).

Both the M(mineral) and protein/fat ratio indicators are positively correlated with deoxyribonucleotide biosynthesis (*P* = 0.025 and *P* = 0.0324) (Fig. 2f) and negatively correlated with polysaccharides biosynthesis (*P* = 0.009 and *P* = 0.006) and chorismate biosynthesis metabolic pathway (*P* = 0.069 and *P* = 0.27), which is responsible for the synthesis of chorismate, a crucial precursor molecule in the production of essential aromatic compounds. Consequently, we observe a negative correlation with the aromatic compounds biosynthesis pathway (*P* = 0.069 and *P* = 0.27).

### Contrasting urban and rural gut microbiome

**Fig 5:**
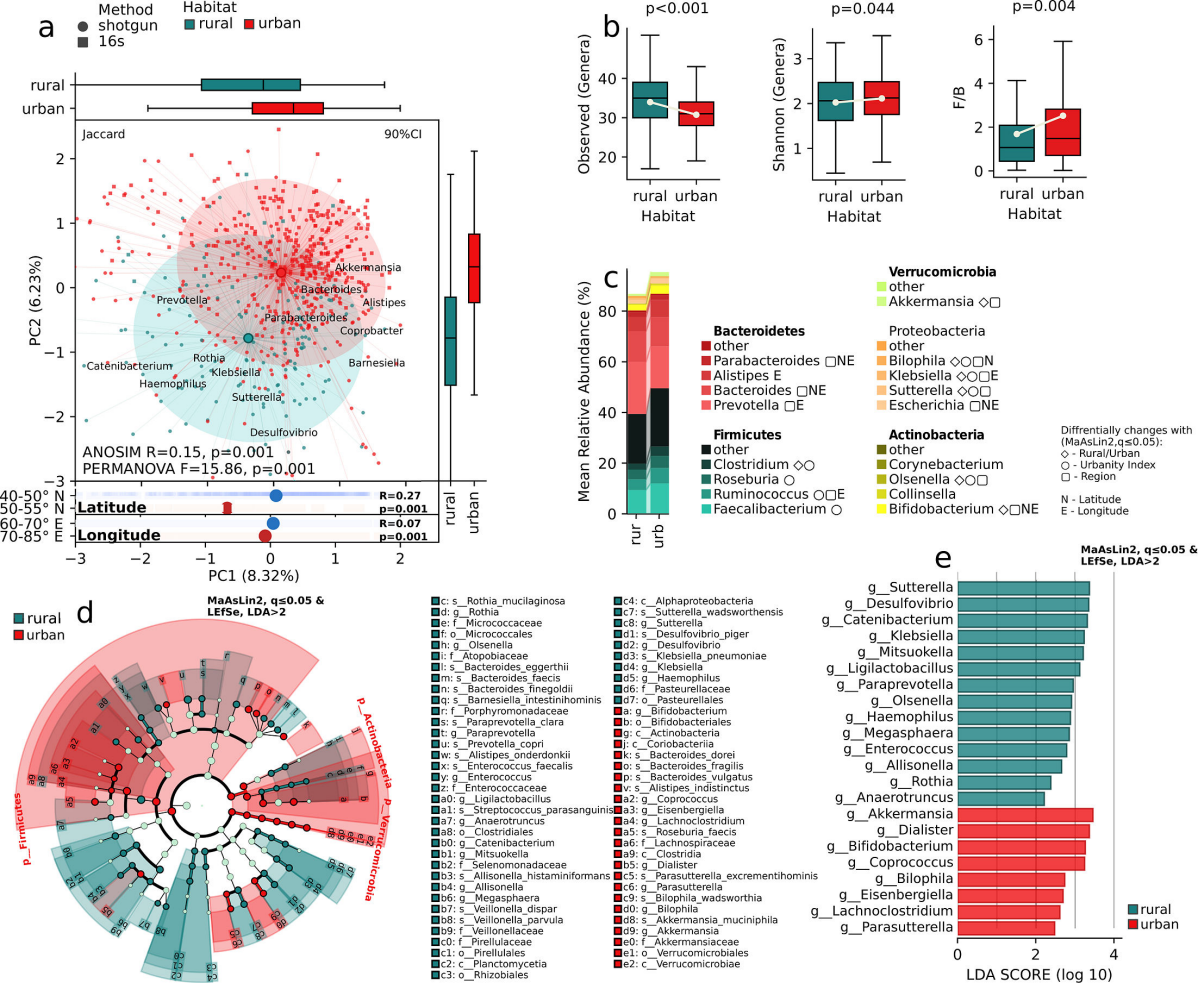
The gut microbiome composition and diversity across rural/urban populations. (a) Principal coordinate analysis plot showing clustering of samples; (b) boxplots showing observed genera richness in rural and urban samples. Observed genera, Shannon index, and Firmicutes/Bacteroidetes ratio. Regression analysis, adjusting for demographic parameters, MaAsLin2, *P* ≤ 0.05; (c) stacked bar plot displaying relative abundances of major bacterial taxa in rural and urban samples. MaAsLin2, *q* ≤0.05, FDR. (d) Cladogram of taxonomic relationships of bacterial groups in rural and urban samples; (e) linear discriminant analysis scores for differentially abundant bacterial taxa in rural and urban settings. MaAsLin2, *q* ≤0.05, FDR, followed by LEfSe effect size analysis, LDA >2.

We have found significant differences in beta (Fig. 5a) and alpha diversity (Fig. 5b) measures between urban and rural populations’ gut microbiomes (*R* = 0.15, *P* = 0.001, *F* = 15.86, *P* = 0.001 and observed, *P* < 0.001, Shannon, *P* = 0.044).

LEfSe analysis combined with MaAsLin2 also confirmed significant differences and allowed us to identify key taxa characteristics of each population (Fig. 5d and e). Dominance of the class, Coriobacteriia*,* Actinobacteria*,* Verrucomicrobiae*,* Clostridia, family Lachnospiraceae*,* Akkermansiaceae, and genera such as *Akkermansia, Bifidobacterium, Bilophila, Coprococcus, Dialister,* and *Parasutterella,* characterizes the gut microbiome composition of urban populations. Conversely, the gut microbiome of rural populations is marked by the prevalence of the class Alphaproteobacteria*,* Planctomycetia*,* and families such as Pasteurellaceae, Pirellulaceae*,* Atopobiiaceae*,* Veillonellaceae*,* Selenomonadaceae along with specific species abundant in rural populations include *Alistipes onderdonkii, Bacteroides eggerthii, Bacteroides faecis, Bacteroides finegoldii, Bacteroides fragilis, Barnesiella intestinihominis, Desulfovibrio piger, Enterococcus faecalis, Klebsiella pneumoniae, Paraprevotella_clara, Prevotella copri,* and *Sutterella wadsworthensis*.

Examination of the dietary data unveiled notable differences in nutritional habits between urban and rural populations across several parameters (Fig. 3a), encompassing NRF14-3 (*P* = 0.046), sodium and chloride intake (*P* = 0.011, *P* = 0.009), mineral intake (*P* = 0.005), total polyunsaturated fatty acids (PUFA) (*P* = 0.014), and higher protein to carbohydrates ratio (*P* = 0.0144).

In an additional analysis comparing rural and urban populations, adjusting for both demographic and dietary variables, some of the taxonomic markers (Fig. 5) appeared to be more associated with dietary variation than urbanity status. Species, *Veillonella parvula*, *Streptococcus parasanguinis*, genera *Ligilactobacillus*, *Enterococcus*, *Mitsuokella*, *Megasphaera*, family Porphyromonadaceae, and orders Rhizobiales and Micrococcales were more strongly associated with salt, cholesterol, NRF14-3, M(vitamins), or saturated fatty acids (SFA) consumption than urbanity status. This trend is reflected in the analysis in Fig. 3e and follows dietary preferences.

The rich diet of urban populations is reflected in the NRF index. The Nutrient-Rich Foods index, indicative of diet quality, shows a positive correlation with the acetyl-CoA-butyrate pathway (*P* = 0.033), pivotal for liver mitochondrial function, and regulated by butyrate, known for its antioxidative effects. Conversely, the NRF index negatively correlates with formaldehyde oxidation (*P* = 0.64), a crucial step in the acetyl-CoA pathway. The protein/fat ratio tends to be higher in urban populations compared to rural counterparts (*P* = 0.18) (Fig S1; Table S2).

**Fig 6:**
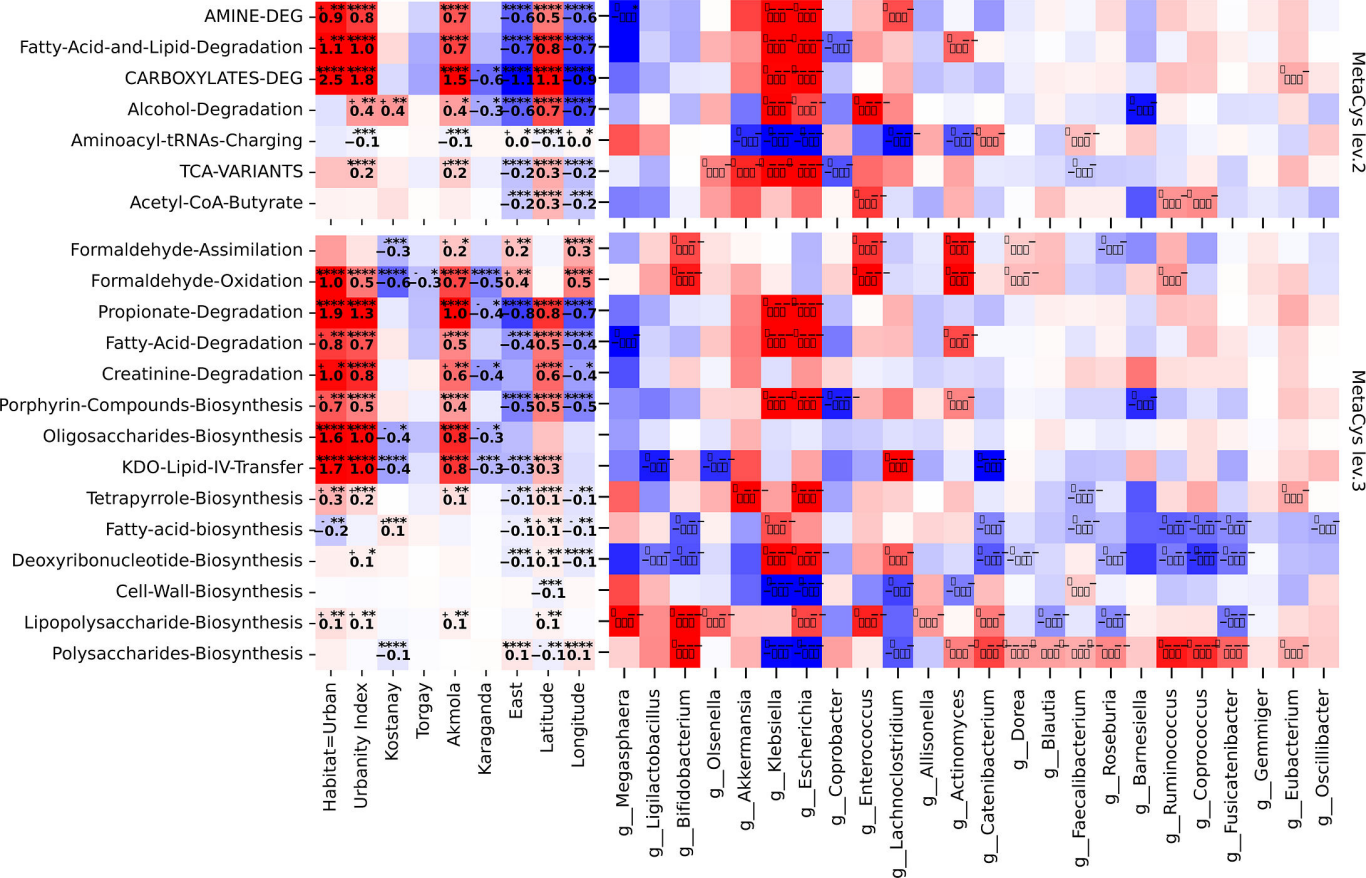
Analysis of associations of nutrition-associated metabolic pathways at levels 2 and 3 of the MetaCys hierarchy, (left) between pathways and location; (right) between pathways and differentially abundant genera (any subgroup), MaAsLin2, *P* ≤ 0.05. Heatmaps display a regression coefficient that indicates the relative strength and direction of the association between variables.

Furthermore, analysis of the gut microbiome and predictive metabolic pathways revealed significant discrepancies between urban and rural settings, notably affecting pathways such as alcohol degradation (*P* = 0.034, association with urbanity index), fatty acid biosynthesis (*P* = 0.001, association with habitat), tetrapyrrole biosynthesis (*P* < 0.001, association with habitat) (Fig. 6).

### Insights into antibiotic resistance and virulence factors

**Fig 7:**
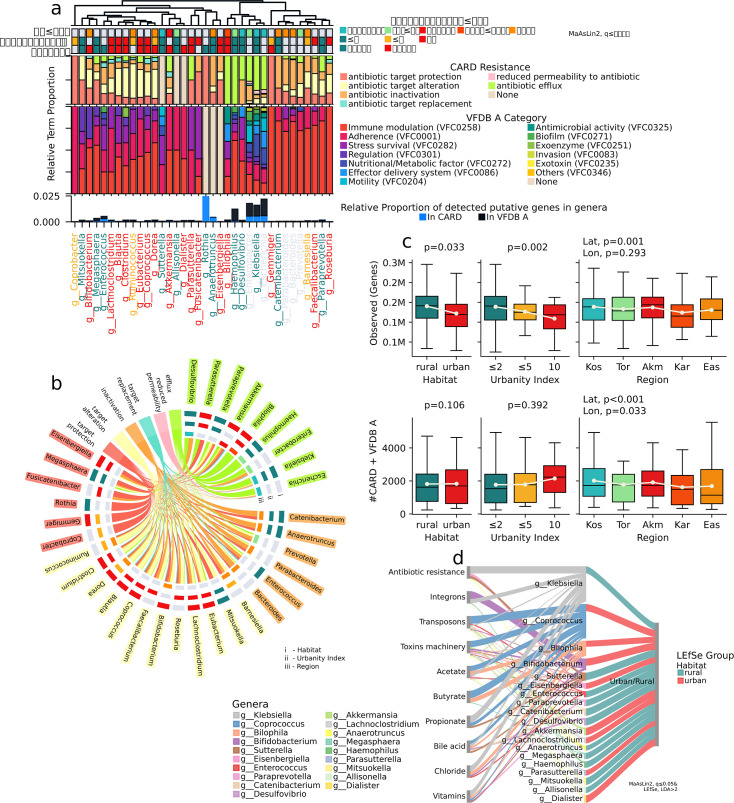
Gut resistome and microbial functions. (a) Dendrogram with bar plots showing the relative proportion of antibiotic resistance genes (CARD database) and virulence factor genes (VF database, set A) detected in differentially abundant genera, in resistance mechanism (CARD annotation) or virulence factor (VFDB annotation) categories. Taxons are clustered based on gene proportions. The heatmap at the top shows the results of the differential analysis and indicates a group with a maximum mean abundance. The bar plot at the bottom shows the relative abundance of the detected genes compared to the total number of genes identified in each genus. (b) Chord plot shows the relative proportion of antibiotic resistance genes in differentially abundant genera, grouped by resistance mechanism (CARD annotation). The heatmap on the edges shows the results of the differential analysis, indicating a group with maximum mean abundance; (c) boxplot displaying the observed gene richness across rurality categories, different urbanity index levels, and geographic locations; (d) displaying the total number of detected CARD and VFDB A genes. MaAsLin2, *P* ≤ 0.05. (d) Flowchart depicting relative gene abundance across several categories (determined using UniProt keyword annotation) in habitat-associated differentially abundant taxa.

To understand the impact of urbanization on microbial gene content, we assessed the presence of potentially beneficial and harmful microbial genes in the gut microbiota of urban and rural populations (Fig. 7a through d). Our investigation centered on harmful genes within the metagenome linked to drug and antibiotic resistance, toxins, pathogenicity islands, and mobile genetic elements, such as integrons and transposons.

In the gut microbiome of the urban population, taxa with mechanisms for replacing or altering the antibiotic action target are often identified while with mechanisms for inactivation in rural population (Fig. 7b). In the rural population, our analysis revealed a predominance of genes associated with toxins machinery (*P* = 0.055, associated with habitat, *P* = 0.006, associated with urbanity index) and mobile genetic elements (*P* = 0.26, associated with habitat, *P* = 0.007, associated with urbanity index), linked to the genus *Klebsiella* among the differentially abundant taxa. Conversely, in the urban population, we observed a higher prevalence of these gene families associated with *Coprococcus* (Fig. 7d).

Interestingly, the proportion of genes associated with antibiotic inactivation is higher in rural areas and regions with lower urbanity indices (≤2) (Fig. S1). On the other hand, the proportion of genes related to virulence factors (VF), such as immune modulation, adherence, stress survival, and regulation, tends to be higher in urban areas and regions with higher urbanity indices (≥10) (Fig. S2; Table S2).

Notably, we detected a significant presence of antibiotic resistance genes (ARGs) associated with tetracycline, glycopeptide, and lincosamide antibiotics. Furthermore, our analysis using the VFDB (Virulence Factor Database) revealed a prevalence of VF-encoding genes associated with stress survival (VFC0282), adherence (VFC0001), regulation (VFC0301), and exoenzyme production (VFC0251). These virulence factors are known to play crucial roles in bacterial survival, colonization, and pathogenesis. Moreover, we found these virulence factors associated with genera *Parasutterella, Mitsuokella, Clostridium, Sutterella, Ruminococcus, Dialister, Enterococcus, Coprococcus,* and *Akkermansia* (>more than 50% of detected genes in these categories) and comprised more than 20% of detected genes in *Bifidobacterium, Megasphaera, Dorea, Bilophila, Desulfovibrio, Parabacteroides,* and *Klebsiella,* underscoring their potential contributions to microbial pathogenicity and host interactions. These genera are known to encompass species with diverse metabolic capabilities and ecological niches, suggesting that the observed virulence factors may contribute to their fitness and persistence within the gut ecosystem.

Among the virulence factors associated with exotoxins in VRDB A, ShET2 (VF0258), colibactin (VF0573), and alpha toxin (CpPLC) (VF0378) were recognized as pivotal contributors to bacterial pathogenicity. In VRDB B, notable virulence factors linked to exotoxins include hemolysin/cytolysin A (VF1134), hemolysin HlyA (VF0646), colibactin (VF1179), hemolysin III (VF0647), colicin-like Usp (VF1135), colicin E1 (VF1182), enterotoxin SenB/TieB (VF1136), colicin Ib (VF1184), colicin Ia (VF1183), and colicin K (VF1186).

## DISCUSSION

We hypothesized that the diversity of the gut microbiome and the prevalence of essential bacterial species undergo significant variations across urbanization, as well as latitude and dietary patterns influencing the gut microbiome. Of particular interest is understanding how these alterations impact the health status of populations and discerning the potential benefits and drawbacks associated with urbanization. Urbanization issues today are acquiring a new level of development. A consequence of urbanization is access to a wide variety of products, and considering the growing prosperity of the urban population, there are more and more high-quality natural products in the food. On the other hand, modern technologies need to be actively introduced in rural areas to increase safety.

The study demonstrated the impact of urbanization on the gut microbiome, highlighting differences in the microbial composition and nutritional patterns of urban and rural populations. Notably, urbanization correlates with altered dietary habits, including increased salt and cholesterol intake but decreased sugar consumption (23–25). WHO studies reveal Kazakhstan has among the highest levels of salt intake globally (26). The NRF index, reflecting diet quality, positively associates with the acetyl-CoA-butyrate pathway, crucial for liver function while negatively correlating with formaldehyde oxidation (27, 28). Urban populations also demonstrate higher protein/fat ratios. Moreover, metabolic pathway analyses reveal significant differences between urban and rural gut microbiomes, impacting pathways such as L-arginine biosynthesis and lipid metabolism.

Urban environments typically have distinct dietary patterns, characterized by high consumption of processed foods, refined sugars, and fats, which may favor the growth of certain bacteria, such as *Akkermansia, Dialister*, and *Bifidobacterium* (29). Conversely, rural areas often have traditional diets rich in fiber, whole grains, and locally sourced produce, promoting the proliferation of bacteria, such as *Mitsuokella, Ligilactobacillus,* and *Paraprevotella* (30–34). At the family level, distinctive prevalence patterns for urban populations exhibit Bifidobacteriaceae and Desulfovibrionaceae, whereas in rural populations families such as Coriobacteriaceae and Veillonellaceae.

Coriobacteriaceae and Bifidobacteriaceae are bacterial families belonging to the Actinobacteria but have different nutritional requirements. Coriobacteriaceae have diverse fermentation profiles and are involved in xenobiotic metabolism (35, 36); they can be influenced by applying a biofertilizer, which increases the content of bioactive compounds in plants (37). In our study, the prevalence of Coriobacteriaceae can be characterized in respondents’ diet by consuming fresh mare’s milk. Our early studies demonstrated that mare’s milk consumption induces growth in members of this family (38). In contrast, the Bifidobacteriaceae family is well known for its probiotic properties and ability to ferment various dietary carbohydrates, including oligosaccharides present in human milk and plant-based foods. Its nutritional requirements and metabolic capabilities differ from Coriobacteriaceae’s, reflecting its distinct ecological niches and functional roles within the gut ecosystem (39).

Also, the gut microbiome of the rural population is characterized by a predominance of the Veillonellaceae family, which is known for its ability to metabolize lactate, a common compound found in fermented milk and fiber-rich foods, which are prevalent in the diet of rural populations in our study (40). Additionally, these bacteria are commonly distributed across various ecological niches, including soil, water, and the gastrointestinal tract of animals. While the characteristic dominance of Desulfovibrionaceae in the gut microbiome of urban populations may be due to diets high in processed foods, fats, and sugars, which may promote the growth of sulfate-reducing bacteria (9, 41, 42).

Environmental factors such as pollution, hygiene practices, and antibiotic use can also influence microbial diversity. Urban areas tend to have higher pollution levels and exposure to antimicrobial agents, which may be selected for bacteria with higher resistance, such as *Coprococcus* and *Bilophila. Bilophila wadsworthia*, a bacterium that reduces sulfate in the human gut microbiome, has significance beyond its association with the foul-smelling hydrogen sulfide gas. While *Bilophila wadsworthia* aids in microbial fermentation and prevents pathogenic colonization in moderation, an overgrowth of this bacterium or an increase in hydrogen sulfide production can cause gut inflammation. This bacterium is linked to a high-fat diet and is associated with several metabolic diseases, including obesity and non-alcoholic fatty liver disease (43, 44). The incidence of these pathologies is on the rise in the regions of Kazakhstan (45, 46).

In contrast, rural environments may harbor bacteria, such as *Klebsiella, Desulfovibrio*, and *Enterococcus*, commonly associated with agricultural practices, livestock exposure, and soil microbiota (47, 48).

*Klebsiella* bacteria utilize carbohydrates, proteins, and fats, favoring simple carbohydrates. In contrast, their ability to metabolize proteins from animal-derived sources, such as meat, poultry, fish, and dairy, supports their growth, relying on vitamin B12 obtained from animal products and fortified grains.

Conversely, we found a positive correlation between the urbanization index and the relative abundance of the genus *Coprococcus*, which exhibits a greater demand for growth-stimulating sources. Various dietary factors, including fiber, polyphenols, resistant starch, prebiotics, butyrate-producing substrates, and certain polyunsaturated fatty acids, can influence the growth of *Coprococcus* bacteria. These bacteria ferment dietary fibers, utilize polyphenols as prebiotics, ferment resistant starch, are stimulated by prebiotic compounds, and produce beneficial short-chain fatty acids like butyrate, potentially contributing to a healthy gut microbiome and overall well-being.

Another undiscussed factor that may influence microbiome composition is altitude. Nevertheless, in the studied region, on average, altitude increases with longitude, which coincides with geographical regions from west to east (from Kostanay to East). Analysis that included geographic, regional, and urbanity status parameters revealed only six associations with altitude. In particular, negative associations were found with the species *Sutterella wadsworthensis*, the genera *Catenibacterium*, and the phylum, class, order, and family of Planctomycetes. There was no association with decrease or increase in diversity and altitude (*P* > 0.05). Notably, the F/B ratio showed a positive significant association with elevation (*P* = 0.0123).

Our research has identified distinct patterns in the prevalence of beneficial and harmful gene families between urban and rural gut microbiomes, consistent with previous studies' findings (49–51). We found that urban environments have slightly higher levels of drug and antibiotic resistance genes (*P* = 0.068), likely due to better healthcare access and higher population density (51, 52). On the other hand, rural populations have higher levels of genes associated with machinery toxins and mobile genetic elements, which may be linked to agricultural practices and poor sanitation (Fig. S2; Table S2) (50, 51, 53).

Our study confirms previous reports that urbanization and environmental factors influence the acquisition and dissemination of resistance genes. In urban areas, the increased use of antibiotics creates selective pressure that favors the growth of resistant microbes (54, 55). In contrast, rural areas promote the propagation of genes that confer resistance to environmental toxins and facilitate horizontal gene transfer (56).

Moreover, our study found variations in the prevalence of virulence factors and antimicrobial resistance genes among specimens. Exotoxin genes such as ShET2, colibactin, and alpha toxin varied among samples, consistent with reports linking these genes to pathogenicity and disease risk (57, 58). Notably, the colibactin-encoding pks gene cluster was associated with specific capsular types and antimicrobial susceptibility patterns, which aligns with previous findings (59, 60). Extended-spectrum β-lactamase genes conferred multidrug resistance, a well-known phenomenon in the literature (61, 62).

Several researchers have shown a close connection between the soil microbiome and the human intestinal microbiome, which arose during evolution and continues to develop (63, 64). All regions in our study differ significantly in basic soil characteristics, such as fertility, organic matter content, and moisture content (65, 66). The dominance of bacteria, such as *Escherichia, Sutterella, Klebsiella, Desulfovibrio*, and *Enterobacter,* is associated with fertile chernozem soils with a high content of organic matter and moisture, which is typical for the Kostanay and Karagandy regions (67). The soils of the Torgay region are of the chestnut type with a low humus content, used as pastures; indeed, in the Torgay region, the population is engaged mainly in livestock farming, and therefore, the intestinal microbiome of the population here is enriched with gut-associated bacteria, such as *Bifidobacterium, Ruminococcus*, and *Bilophila*, which are more often found in the intestines of humans and animals (66).

These findings highlight the complex interplay between urbanization, environmental exposures, and acquiring beneficial and harmful gene repertoires within the gut microbiome.

### Study limitations

Our study on the effects of urbanization on gut microbiome composition and dietary patterns has some limitations that need to be acknowledged. First, our findings are based on observational and cross-sectional data spanning a large geographic area with a variety of potentially confounding interactions, including geographic and regional variation, which hinders the attempts to firmly assert and disentangle casual associations between urbanization, nutritional factors, and gut microbiome changes. Second, there may be selection bias in our study as participants were recruited from specific regions and may not fully represent the diversity of urban and rural populations. Third, dietary data were self-reported, which may introduce the potential for recall bias and inaccuracies. Furthermore, we used two different methods for gut microbiome analysis—16S sequencing and shotgun sequencing—which may introduce errors in the analysis. Additionally, while we tried to control for variables such as age, gender and BMI, there may be other unmeasured factors that could affect gut microbiome composition and dietary habits in our study. Lastly, the study focused on a specific geographic region, which may limit the generalizability of our findings to other populations with different cultural and environmental contexts.

### Conclusion

This study explores how urbanization can potentially shape the gut microbiome’s mosaic across Kazakhstan’s geographical and dietary landscapes. The findings underscore the intricate interplay between environmental exposures, dietary lifestyles, and the microbial residents that inhabit our intestines. Urban environments were associated with reduced microbial diversity, an elevated Firmicutes/Bacteroidetes ratio, and an increased prevalence of potentially harmful genera, while rural populations exhibited greater diversity and abundance of beneficial bacteria. Importantly, the study also revealed distinct patterns in the prevalence of ARGs and virulence factors between urban and rural gut microbiomes, suggesting the complex relationship between urbanization, environmental factors, and the acquisition of both beneficial and harmful microbial genes.

## MATERIALS AND METHODS

### Participants

The prospective cohort study investigates the gut microbiome of urban/rural health in Kazakhstan. All participants provided informed consent. The study included 651 respondents from Astana city, Karagandy city, Kostanay, Torgay (Akmola region), and Ridder (East Kazakhstan region), as well as small villages near the listed cities and towns.(Table 1) The sample comprised 502 urban and 149 rural participants, with 503 (77.3%) females and 148 (22.7%) males. When selecting settlements, we were guided by the selection of villages that were as remote as possible from main roads, in the food assortment that did not have fast food products, carbonated sweet drinks, the cuisine of populations mainly consists of natural products as well as the urban population from the city/town closest to this rural settlement. The age of the study participants ranged from 22 to 101 years (52.5 ± 16.2). The average age of urban residents was 51.6 ± 17.3 years, while the average age of rural residents was 56.6 ± 9.3 years. All settlements were stratified into groups according to the published urbanization index (68).

**TABLE 1:** Geographical distribution of participants

Locality	Latitude	Longitude	*n* Participants	Urban/rural
Kokshetau	53.298	69.326	1	Urban
Kostanay	53.206	63.457	28	Urban
Amankonyr	51.84	73.384	2	Rural
Torgay	51.758	72.728	13	Rural
Ereymentau	51.506	69.333	1	Urban
Zhansary	51.166	73.67	31	Rural
Astana	51.032	71.483	361	Urban
Novodolinka	50.848	71.938	3	Rural
Semey	50.413	80.193	1	Urban
Ridder	50.34	83.42	13	Urban
Rodina	50.277	66.87	30	Rural
Temirtau	50.067	72.868	34	Urban
Karagandy	49.824	72.839	90	Urban
Saran	49.804	72.751	5	Rural
Shakhtinsk	49.717	72.54	2	Rural
Abay	49.632	72.861	4	Rural
Karkaraly	49.411	75.458	2	Rural
Akzhar	47.578	83.674	26	Rural
Balhash	46.85	74.889	3	Rural
Ushtobe	45.25	77.94	1	Rural

### Data collection

Participants completed a paper questionnaire on demographic characteristics, health status, and FFQ (Food Frequency Questionnaire). Stool samples for microbiome studies were collected in DNA/RNA Shield Fecal Collection Tube (Zymo Research, R1101). All samples were stored in a refrigerator at +4°C until DNA extraction.

### Diet study

To analyze the diet, we conducted a survey using the FFQ questionnaire. All responses were compiled for each participant and uploaded to the FFQ EPIC Tool for Analysis, available at https://www.epic-norfolk.org.uk/for-researchers/ffq/. For further analysis, we calculated: (i) macronutrients ratio, (ii) NRF (69), and (iii) average of individual ratios.

- Macronutrient ratio is the ratio of the amount of protein to fat, carbohydrates, and carbohydrates to fat in grams—protein/fat g, protein/carbohydrate g, and carbohydrate/fat g.
- NRF index is calculated as the sum of the ratios of beneficial nutrients to the reference value (per 2,000 kcal)—NR (nutrients to be encouraged) minus the sum of the ratios of “harmful” nutrients to the reference value (per 2,000 kcal)—LIM (nutrients to be limited). NR includes Englyst Fiber—non-starch polysaccharides; protein; vitamin A—retinol; vitamin B12—cobalamin; vitamin C—ascorbic acid; vitamin D—ergocalciferol; vitamin E—alpha-tocopherol equivalents; total folate; calcium; iron; potassium; magnesium; monounsaturated fatty acids (total); PUFA (total); milk and milk products; nuts and seeds; fruit; vegetables. LIM includes SFA (total), sodium mg + chloride, sugars, preserves, and snacks.
- The average of individual ratios is M(macronutrients) values. M is the average of the ratios of nutrients to the reference—the closer to 1, the closer the entire category is to the reference. Similarly, on average, above 1—use is higher than the reference, less than 1—below the reference. Reference values of macro- and micronutrients are determined according to earlier publications (69).

### Metagenomic research

DNA was extracted using ZymoBiomics DNA Microprep (Cat. No: D4300), and DNA concentrations were measured using Nanodrop 2000/2000c (ThermoFisher). Sterile water served as a negative control. Following the standard Illumina protocols, the V3-V4 region of 16S loci was sequenced at Novogene (Beijing, China) on the Illumina NovaSeq6000 platform.

### Statistical analysis

Statistical analysis was performed using Python v3.9.16 and R v4.2.2. Taxonomic data consisted of WGS (*n* = 351) and 16S amplicon (*n* = 301) sequencing data. WGS data processed by MetaPhlAn were normalized to relative abundance. 16S OTU tables were normalized using TSS. Taxons from WGS and 16S tables were merged on taxon naming. To account for shift in abundance distribution in 16S data, we performed QQ normalization. Subsequently, we applied MMUPHin 1.12.1 batch adjustment to correct for technical variation and to adjust for covariates. The merged data were filtered to remove low prevalence taxa (<13%).

For pathway analysis, we used a subset of WGS (*n* = 199) and 16S (*n* = 193) data. MMUPHin was used to adjust for technical variation like in taxonomic data. WGS functional data were further used for gene family composition analysis. EggNOG-mapper v2 was used to infer KEGG annotation. Additionally, we aligned data to the VFDB (set A and B) and CARD databases (homolog model) using diamond v2.1.9.163 (bitscore >60, identity >60%, minimum alignment length >30 AA).

Nutrition data (*n* = 600) was normalized to 2,000 kcal. The NRF was calculated as in reference (69).

Beta ordination was performed using scikit-bio v0.5.6 on Hellinger-transformed data using unweighted and weighted UniFrac, Jaccard, and Bray-Curtis distances. Quality of separation was tested using scikit-bio. Alpha diversity was assessed using observed and Shannon indices. Enterotyping was performed according to the protocol of Arumugam et al. (70).

For differential analysis, we used MaAsLin2 v1.12.0 in combination with LEfSe v1.1.2 analysis. We used MaAsLin2 to find taxonomic differentially abundant features while correcting for demographic characteristics (*q* ≤0.05, FDR threshold). Then, we performed LEfSe analysis with significance threshold LDA ≥2 and *P* ≤ 0.05. Associations between selected nutrients, functional features, and differentially abundant taxa were then determined using MaAsLin2, correcting for demographic characteristics without applying correction for multiple comparisons (*P* ≤ 0.05).

Visualization was produced using Matplotlib v3.7.1 and seaborn v0.11.2 libraries. Chord plots were generated using PyCircos v0.3.0, Sankey diagrams were generated using SankeyFlow v0.3.8, and statistical annotations were placed using statannotations v0.5.0.

## Data Availability

The raw data of 16S rDNA sequencing and shotgun metagenomic sequencing have been deposited to NCBI under the following BioProject ID: PRJNA1124098 and PRJNA1048722. The lead contact can provide any additional information required to reanalyze the data reported in this paper upon request. The underlying code for this study is available in GitHub and can be accessed via this link: https://github.com/VeaLi/kgeography2024.

## AFTER EPUB

*[This article was published on 17 September 2024 with an error in Acknowledgments. The Acknowledgments were corrected in the current version, posted on 10 October 2024.]*

## SUPPLEMENTAL MATERIAL

Fig. S1 msystems.00585-24-s0001.eps A comprehensive analysis of the relative proportion of various nutrient components across different factors.

Fig. S2 msystems.00585-24-s0002.eps A comprehensive analysis of the relative proportion of putative genes detected in different data sets.

Legends msystems.00585-24-s0003.docx Legends for supplemental figures.

Table S1 msystems.00585-24-s0004.xlsx Regression coefficients and *P*-values for the association between dietary factors and habitat/geographic variables.

Table S2 msystems.00585-24-s0005.xlsx Regression coefficients and *P*-values for the association between antibiotic resistance genes/mobile genetic elements and habitat/geographic variables.

ASM does not own the copyrights to Supplemental Material that may be linked to, or accessed through, an article. The authors have granted ASM a non-exclusive, world-wide license to publish the Supplemental Material files. Please contact the corresponding author directly for reuse.
