# Supplementary material for: Impact of urbanization on gut microbiome mosaics across geographic and dietary contexts
Source: mSystems. 2024 Sep 17;9(10):e00585-24. doi: 10.1128/msystems.00585-24 (PMC11494887; doi:10.1128/msystems.00585-24)
Supplement: Legends — for supplemental figures. [file msystems.00585-24-s0003.docx]

Figure 1S. A comprehensive analysis of the relative proportion of various nutrient components across different factors like rural/urban areas, an urbanity index, geographical regions, latitude, and longitude: M(Minerals), Protein/Carbs, Sodium, Chloride, PUFA, NRF-14, MUFA, Protein/Fat, Cholesterol, SFA, M(vitamins).

Figure 2S. A comprehensive analysis of the relative proportion of putative genes detected in different datasets: across various factors such as rural/urban areas, an urbanity index, geographical regions, latitude, and longitude: Integrons, Observed (Genes), Toxins, Mobile Elements, Transposons, CARD (Comprehensive Antibiotic Resistance Database) and VFDB A (Virulence Factors of Bacterial Pathogens).
